# Cartilage Conduction Hearing and Its Clinical Application

**DOI:** 10.3390/audiolres11020023

**Published:** 2021-06-03

**Authors:** Tadashi Nishimura, Hiroshi Hosoi, Ryota Shimokura, Chihiro Morimoto, Tadashi Kitahara

**Affiliations:** 1 Department of Otolaryngology-Head and Neck Surgery, Nara Medical University, 840 Shijo-cho, Kashihara, Nara 634-8522, Japan; mori-chi@naramed-u.ac.jp (C.M.); tkitahara@naramed-u.ac.jp (T.K.); 2 MBT (Medicine-Based Town) Institute, Nara Medical University, 840 Shijo-cho, Kashihara, Nara 634-8522, Japan; hosoi@naramed-u.ac.jp; 3 Graduate School of Engineering Science, Osaka University, D436, 1-3 Machikaneyama, Toyonaka, Osaka 560-8531, Japan; rshimo@sys.es.osaka-u.ac.jp

**Keywords:** cartilage conduction, airborne sound, aural atresia, hearing aid, bone conduction, bone-anchored hearing aid, conductive hearing loss

## Abstract

Cartilage conduction (CC) is a form of conduction that allows a relatively loud sound to be audible when a transducer is placed on the aural cartilage. The CC transmission mechanism has gradually been elucidated, allowing for the development of CC hearing aids (CC-HAs), which are clinically available in Japan. However, CC is still not fully understood. This review summarizes previous CC reports to facilitate its understanding. Concerning the transmission mechanism, the sound pressure level in the ear canal was found to increase when the transducer was attached to the aural cartilage, compared to an unattached condition. Further, inserting an earplug and injecting water into the ear canal shifted the CC threshold, indicating the considerable influence of cartilage–air conduction on the transmission. In CC, the aural cartilage resembles the movable plate of a vibration speaker. This unique transduction mechanism is responsible for the CC characteristics. In terms of clinical applications, CC-HAs are a good option for patients with aural atresia, despite inferior signal transmission compared to bone conduction in bony atretic ears. The advantages of CC, namely comfort, stable fixation, esthetics, and non-invasiveness, facilitate its clinical use.

## 1. Introduction

The sound transmission pathway to the cochlea is generally classified into air and bone conduction (AC and BC). For AC, sound generated outside the ear travels to the eardrum through the ear canal and is transduced into vibrations of the ossicles to reach the cochlea. For BC, skull bone vibrations induced by a transducer are transmitted to the cochlea, involving at least five components [1,2,3]. Sound can also be perceived by body part vibrations other than the skull bone [4,5,6], and the transmission mechanisms are unique from one another. When the transducer is placed on the aural cartilage, particularly on the tragus, a relatively loud sound is audible [7]. This form of conduction is referred to as cartilage conduction (CC) [8]. Generally, hearing through non-osseous soft tissue conduction is not as clear as conventional BC. However, a clear sound is audible in CC, and it is perceived louder than when a transducer is placed on the mastoid or forehead [9].

The hypothesized CC mechanism is different from AC and BC [10,11]. For a vibration speaker, the sound signal increases by a movable plate, and the amplified signal is transmitted via AC. For CC, the vibration of the cartilaginous portion of the ear canal induced by a transducer generates sound in the ear canal. In this transduction, the cartilaginous portion of the ear canal functions like the movable plate of a vibration speaker, and thus the signal in the ear canal increases in amplitude compared to when the transducer is unattached to the aural cartilage. The airborne sound in the canal is subsequently transmitted via the eardrum in the same manner as with AC. The signal is predominately transmitted via the eardrum and ossicles, although CC delivers the signals by vibrating a transducer, similar to BC or non-osseous BC. Therefore, the conduction characteristics resemble AC rather than BC. In contrast to AC, CC uses the aural cartilage in the same way as the moveable plate of a vibration speaker to generate airborne sound. In other words, a part of the human body (aural cartilage) contributes to airborne sound generation. This hypothesis underlying the generation of airborne sound in CC is unique and currently not fully understood. Due to the unique characteristics of CC, acoustic devices utilizing CC may potentially provide benefits that cannot be obtained with AC or BC devices. To develop CC devices further, the mechanism underlying the conduction must be established. With this review, we aim to summarize previous reports regarding CC that we found on PubMed (search term “cartilage conduction hearing”) to facilitate its understanding.

## 2. The Theoretical CC Transmission Pathway

**Figure 1:**
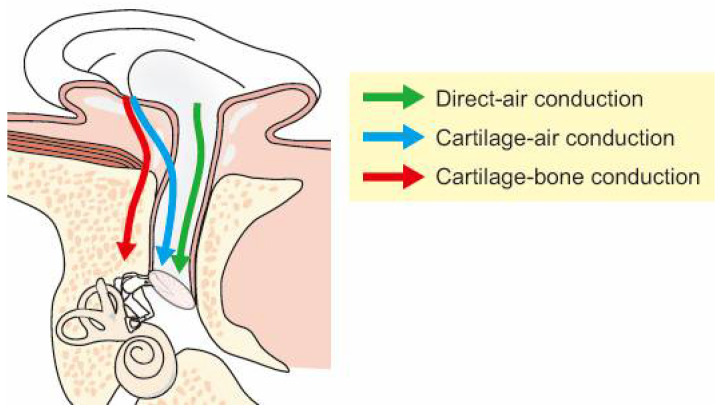
Possible cartilage conduction pathways. (Figure 1 was originally presented in Nishimura et al. 2015, Figure 1 [11]).

There are three possible transmission pathways when a transducer is placed on the aural cartilage, as presented in Figure 1 [10,11]. In the first pathway, transducer vibrations directly produce airborne sound, some of which reach the ear canal and are transmitted to the cochlea via the conventional AC pathway. This pathway is termed “direct-AC” and has the same transduction mechanism as AC. In the second pathway, aural cartilage vibrations are transmitted to the cartilaginous portion of the ear canal. These vibrations induce an acoustic signal in the canal that reaches the eardrum, transmitted via the ossicles. This pathway, which uses the aural cartilage as a movable plate, is termed “cartilage-AC” and is a transduction mechanism different from those of AC and BC. In the third pathway, aural cartilage vibrations are transmitted via the skull. This pathway is termed “cartilage-BC,” and is considered similar to BC because the delivered mechanical signal is directly transmitted via the skull bone.

## 3. Sound Pressure Level in the Ear Canal via CC

**Figure 2:**
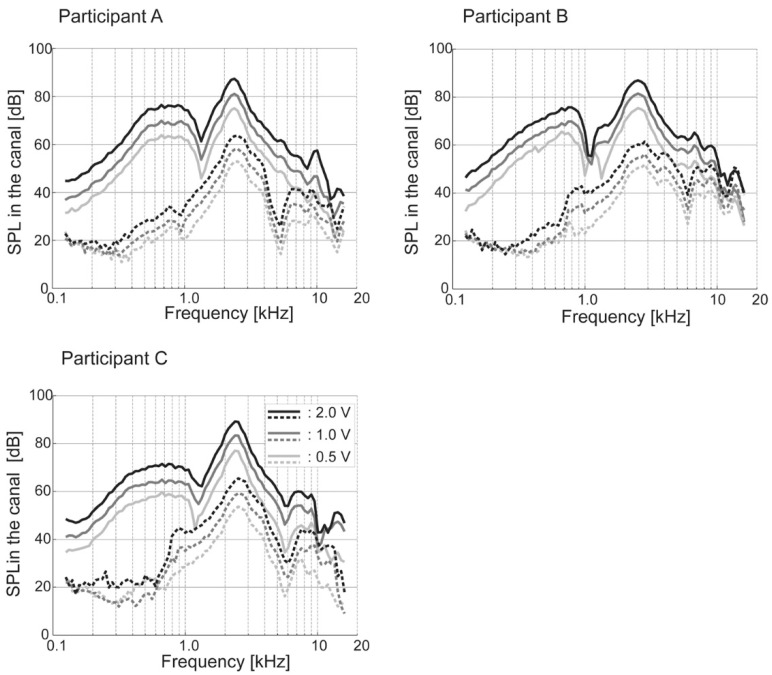
Sound pressure level (SPL) in the canal when the transducer is attached to the tragus (—) and unattached (- - -). The black, dark gray, and gray lines indicate input voltages of 2.0, 1.0, 0.5 V, respectively. (Figure 2 was originally presented in Shimokura et al. 2014, Figure 6 [13]).

A loud sound is audible when a transducer is attached to the aural cartilage. There are no standard evaluation methods for CC hearing. The measurement of the sound level in the ear canal similar to real-ear measurements [12] contributes towards understanding the phenomenon. Shimokura et al. objectively demonstrated the loudness increase by measuring the sound pressure level in the ear canal using a probe microphone (Figure 2) [13]. The sound pressure level in the ear canal improved when the transducer was attached to the aural cartilage compared to the unattached condition in all participants. The improvements from the attached condition were largest at low to mid frequencies, with gains reaching approximately 40 dB at frequencies between 500 Hz and 1000 Hz. Conversely, to reproduce the difference in the sound pressure level in the ear canal between the attached and unattached conditions, not only the bony portion of the ear canal but also the cartilaginous portion was necessary to consider [14]. The airborne sound generated by a simulated cartilaginous portion (movable plate) played an important role in the reproduction of the sound pressure level in a simulated ear canal. These findings suggest the predominance of the cartilage-AC pathway in CC in the attached condition.

## 4. Hearing Threshold Measurements via CC

### 4.1. Threshold Shift with an Earplug

In a previous study, an earplug was used to show differences in the characteristics between CC and AC or BC [9]. Thresholds with and without the earplug were measured at 500–4000 Hz using a transformed up-down procedure (two-alternative forced-choice) [15]. The earplug interferes with both AC and direct-AC in CC. For AC, the thresholds worsened with the earplug for all frequencies. For CC, the threshold worsened with the earplug above 2 kHz, but the thresholds at low to mid frequencies did not; they were stable at 1000 Hz and improved at 500 Hz. These observations demonstrate that direct-AC is not the predominant pathway in CC. Furthermore, for BC the thresholds at mid to high frequencies were stable with the earplug, which also disagreed with the CC results.

A transducer can be placed in various ways on the aural cartilage. Another study evaluated the effect of an earplug on the thresholds when a transducer without a static force was placed on the tragus, soft tissue (pre-tragus region), and mastoid [16]. Thresholds with and without the earplug were measured at 500–4000 Hz using a transformed up-down procedure [15]. The thresholds for the tragus placement were significantly better than for other placements, both with and without the earplug, except with the earplug at 4000 Hz. The threshold elevations with the earplug for the tragus placement were significantly larger than those for the mastoid placement at 2000 and 4000 Hz. These results demonstrate that placing the transducer on the aural cartilage contributes to hearing improvement. Low-frequency boost can influence speech perception. Although there was no deterioration in speech recognition in the open ear, excessive low-frequency boost in the occluded condition reduced the scores, even in individuals with normal hearing [17]. Frequency adjustment may be necessary for the occluded ear when excessive low-frequency boost deteriorates speech perception [18].

### 4.2. Threshold Shift with Water Injected into the Ear Canal

**Figure 3:**
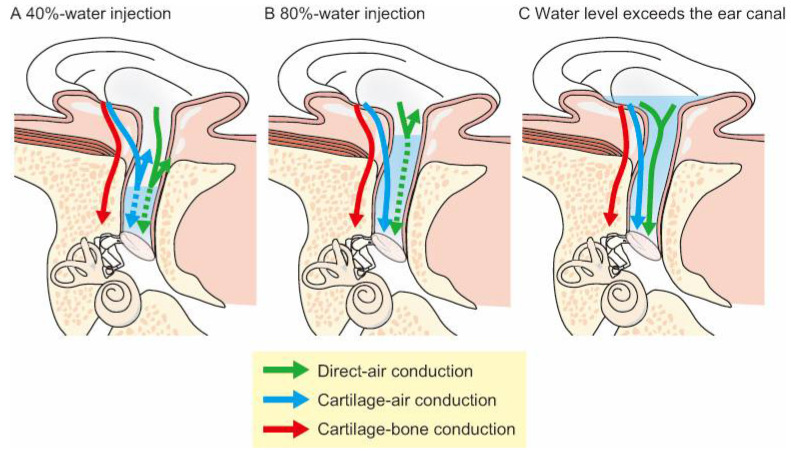
Effects of ear canal water injection on the transmission pathways. (**A**) The water stays within the bony portion of the ear canal, interrupting direct- and cartilage–air conduction. (**B**) The water enters the cartilaginous portion of the ear canal, avoiding an impedance mismatch between air and water in the cartilage-AC pathway. (**C**) The water level exceeds the ear canal, allowing for direct water vibrations. (Figure 3 was originally presented in Nishimura et al. 2015, Figure 1 [11]).

Previous studies using earplugs have contributed to establishing the conduction mechanism of CC [9,16,17]. Earplugs generate an occlusion effect, which influences low-frequency thresholds. Thus, previous studies used ear canal water injections instead of earplugs to avoid the occlusion effect [11]. AC, BC, and CC thresholds were measured at 500–4000 Hz with water injected into the ear canal using a transformed up-down procedure [15]. To measure the thresholds in the water-injected condition, subjects laid on a bed in a lateral recumbent position with the entrance of the ear canal facing the ceiling and the head fixed to avoid water fluctuations in the canal. Figure 3 illustrates the influence of water injections on three theoretical CC components. If the cartilaginous portion vibrations are dominant, the thresholds will increase when the water stays within the bony portion of the ear canal (Figure 3A), and then decrease when the water reaches the cartilaginous portion (Figure 3B). If the threshold improves when the water level is so high that it reaches the transducer (Figure 3C), then transmission through the cartilaginous portion is likely not the dominant pathway. Thus, the relationship between the threshold and water volume demonstrates the relative contribution of the three possible pathways to CC. The results of these studies revealed that injecting water into the ear canal elevated the AC thresholds by 22.6–53.3 dB, and the threshold shifts for BC were within 14.9 dB [11]. For CC, when the water was within the bony portion of the ear canal (i.e., 40% of the ear canal length in the previous study; Figure 3A), the thresholds were elevated by the same degree as AC. When the water line reached the cartilaginous portion (i.e., 80% of the ear canal length in the previous study; Figure 3B), the thresholds at 500 and 1000 Hz decreased by 27.4 and 27.5 dB, respectively. Additionally, despite blocking the ear canal with water, the force levels of the CC transducer at the thresholds measured with an artificial mastoid were clearly lower than those of the BC transducer at the threshold. The vibrations of the cartilaginous portion contributed to sound transmission, particularly in the low-frequency range. Although the airborne sound radiates into the ear canal in BC and CC, the generation mechanisms are different. CC generates airborne sounds in the canal more efficiently than BC.

The effect of water in the ear canal was also evaluated at 500–2000 Hz for five different placements of the transducer: the tragus, intertragal incisure, anti-tragus, pre-tragus, and mastoid [19]. Among the CC conditions (tragus, intertragal incisure, and anti-tragus), the results showed the same amount of threshold shifts when water was injected into the ear canal, and the fixation placement did not affect the threshold shifts by water injection. Thus, the cartilage-AC characterizes the acoustic properties of CC.

## 5. CC in Pathological Ears

The transmission pathway or mechanism may change in pathological ears, e.g., the atretic ear whose condition is quite different from that of the normal ear. In the bony atretic ear, the AC route is not present, and most signals are transmitted to the cochlea via the skull bone. For CC, cartilage-BC is considered the predominant pathway instead of direct- and cartilage-AC (Figure 1) in the bony atretic ear. The impedance mismatch between the soft tissue and skull bone obstructs transmission. As the transducer is placed without a static force, CC and AC do not have conduction efficacy advantages over BC. Conversely, the transmission conditions in ears with fibrotic aural atresia are quite different. Vibrations are transmitted to the cochlea via fibrotic tissues instead of the skull bone. This fibrotic pathway allows the signals to travel to the cochlea, avoiding the large impedance mismatch between the soft tissue and skull bone. Some patients with fibrotic aural atresia have much better thresholds with CC (30–50 dB at low frequency compared to BC) [20]. Hence, CC has a transmission advantage over BC in the case of the fibrotic pathway.

## 6. CC Applications

**Figure 4:**
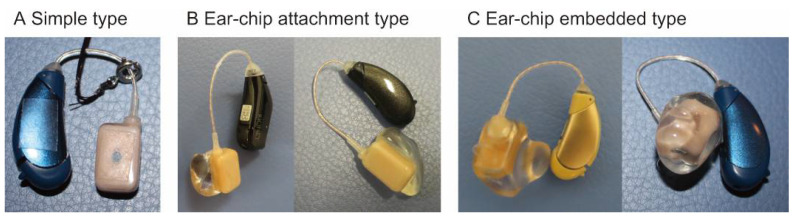
Cartilage conduction hearing aids (HB-J1CC, Rion Co Ltd., Kokubunji, Tokyo, Japan) have three transducer types: (**A**) simple-attachment, (**B**) ear-chip attachment, and (**C**) ear-chip embedded.

Acoustic devices that utilize CC, including smartphones and hearing aids, have been developed [8,21,22,23]. CC hearing aids (HA; CC-HA) have already been used in clinical practice in Japan since 2017. When direct- and cartilage-AC are functional (such as for sensorineural hearing loss), a commercially available CC-HA (Figure 4) could provide adequate amplification for mild to moderate hearing loss, as estimated by measuring the output level using a simulator which can evaluate the airborne sound in CC [24]. When direct- and cartilage-AC are not functional, patients who receive the most benefits from CC-HAs are patients with aural atresia. These patients require BC-HAs or implantable devices to achieve sufficient amplification [25,26,27,28,29,30,31]. However, conventional BC-HAs have disadvantages associated with their fixation style; the transducer is fixed with a headband using static force, which can lead to discomfort, pain, and irritation [26]. The fixation of the transducer can cause poor esthetics. Surgical procedures, such as implanting bone-anchored hearing aids (BAHAs), are additional options [25,26,27,28,29,30,31] but involve various risks, such as adverse medical and surgical events, infection, and follow-up surgery [32,33]. Some patients also refuse BAHA implantation because of cosmetic considerations [34]. In contrast, the CC transducer is fixed without a static force, mitigating some of the fixation problems with BC-HAs, and it does not require surgery. In contrast to AC, CC mechanical signals can be delivered directly to the tissue. CC also has transmission advantages in the atretic ear over AC because it avoids the impedance mismatch between air and skin. Thus, CC-HAs are a possible alternative for patients with aural atresia.

### 6.1. CC-HA Characteristics

**Figure 5:**
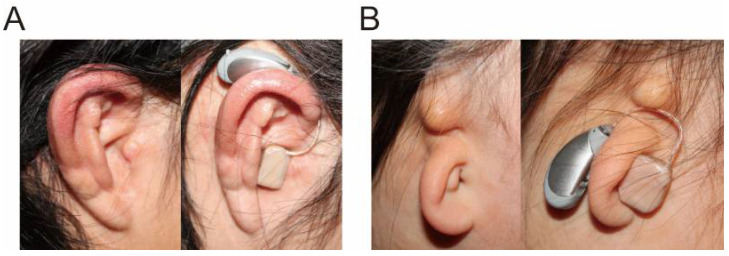
Examples of ears with and without cartilage conduction hearing aid (CC-HA). Some patients wear CC-HA in the same manner as conventional behind-the-ear hearing aids (**A**). For other patients, double-sided tape is needed for fixation of the hearing aids (**B**). (Figure 5 was originally presented in Nishimura et al. 2018, Figure 1 [40]).

CC-HAs are behind-the-ear HAs (Figure 4), with the transducer placed on the aural cartilage and the signal delivered through the cartilaginous tissue [35]. The transducer, optimized to transmit vibrations to the aural cartilage, is small and lightweight (11.9 × 7.8 × 4.7 mm, 1.4 g). It is easily attached to the ear because of the conchal cartilage stiffness, even when only a small cavity is present on the ear surface (Figure 5A). In the absence of a sufficiently large cavity, CC-HA transducers can be attached with double-sided tape (Figure 5B). Therefore, neither a high contact pressure nor a headband is required for attachment. There is little risk of skin irritation, as experienced by patients who use conventional BC-HAs, or infection, as experienced by patients with implanted BAHAs [36,37], and they can be used from infancy. In Japan, CC-HA has become an option for treating atretic ears. The Oto-Rhino-Laryngological Society of Japan puts the information related to CC hearing aids along with that related to BAHAs, Vibrant Soundbridge (VSB), and cochlea implants at its website [38], and the guidelines for implantable devices such as BAHAs, VSB and Bonebridge authorized by the Japan Otological Society [39] require CC hearing aids to be tested before the decision of their indication.

### 6.2. CC-HA Benefits

CC-HAs were first reported in 2010 [21], and benefits for patients with chronic otitis media and aural atresia were reported in 2013 [22]. A clinical study with 41 patients (21 with bilateral aural atresia, 15 with unilateral aural atresia, and five with other diseases) demonstrated that CC-HAs can provide audiometric benefits equivalent to those of other devices (AC-HAs, BC-HAs, and BAHAs) without any serious adverse effects [40]. After the trial, 95% and 93% of the patients with bilateral and unilateral aural atresia, respectively, continued using their CC-HAs. Most patients who tried CC-HAs reported improvements in communication abilities in noisy environments and sound localization. Another study reported that laterality judgements significantly improved in bilateral aural atresia patients with CC-HAs [41]. Sakamoto et al. evaluated CC-HA benefits in patients with unilateral congenital atretic ears [42] and reported that speech recognition scores improved in a noisy environment. Nishiyama et al. investigated adult candidates for CC-HA treatment [43] and concluded that patients with ear canal stenosis or atretic ears were the most suited candidates. They also reported good outcomes in children with the same ear conditions [44]. To investigate the clinical use of CC-HAs in Japan, a survey was performed in nine medical institutions with 256 patients who tried CC-HAs [35]. Similar to previous studies, the survey demonstrated that the candidates for CC-HAs were patients with aural atresia. Sixty-five patients with bilaterally and 124 patients with unilaterally closed ears (aural atresia or severe canal stenosis) tried CC-HA use. The purchase rate after the trial was 86% and 78%, respectively, for these two groups of patients. Patients with refractory continuous otorrhea who experienced difficulties with AC-HA use also showed a high purchase rate (78%). In contrast, the purchase rate for patients who had no difficulty with AC-HA use, such as patients with sensorineural hearing loss, was significantly lower (37%). Finally, there were no differences between the CC-HAs and the patients’ own hearing devices regarding audiometric results in the atretic ears, such as aided threshold, functional gain, and speech recognition [34,40]. Even though CC transmission is inferior to BC transmission in bony atretic ears, the audiometric outcomes were comparable [35,40], and other advantages, such as comfort, stable fixation, cosmetics, and non-invasiveness, may explain the high acceptance.

### 6.3. Limitations

CC-HAs have only been used in clinical practice since 2017, which is not long enough to thoroughly establish their indication criteria, fitting technique, and benefits. Furthermore, comparisons between CC-HAs and implantable devices have not been performed yet. Further investigations are therefore required for establishing CC-HAs in clinical practice.

## 7. Conclusions

In CC, the aural cartilage plays a similar role to the movable plate of a vibration speaker. This transduction mechanism, unique from AC and BC, is responsible for the CC characteristics. CC can be applied to various acoustic devices, and there have been rapid advances in HA development using CC. CC-HAs can be a beneficial option for patients with aural atresia, although CC does not always surpass BC in terms of transmission efficacy in bony atretic ears.

## Data Availability

Not applicable.
